# Genome-wide comparative analysis of microRNAs in three non-human primates

**DOI:** 10.1186/1756-0500-3-64

**Published:** 2010-03-09

**Authors:** Markus Brameier

**Affiliations:** 1 Primate Genetics Laboratory, German Primate Center, Leibniz Institute for Primate Research, D-37077 Göttingen, Germany

## Abstract

**Background:**

MicroRNAs (miRNAs) are negative regulators of gene expression in multicellular eukaryotes. With the recently completed sequencing of three primate genomes, the study of miRNA evolution within the primate lineage has only begun and may be expected to provide the genetic and molecular explanations for many phenotypic differences between human and non-human primates.

**Findings:**

We scanned all three genomes of non-human primates, including chimpanzee (*Pan troglodytes*), orangutan (*Pongo pygmaeus*), and rhesus monkey (*Macaca mulatta*), for homologs of human miRNA genes. Besides sequence homology analysis, our comparative method relies on various postprocessing filters to verify other features of miRNAs, including, in particular, their precursor structure or their occurrence (prediction) in other primate genomes. Our study allows direct comparisons between the different species in terms of their miRNA repertoire, their evolutionary distance to human, the effects of filters, as well as the identification of common and species-specific miRNAs in the primate lineage. More than 500 novel putative miRNA genes have been discovered in orangutan that show at least 85 percent identity in precursor sequence. Only about 40 percent are found to be 100 percent identical with their human ortholog.

**Conclusion:**

Homologs of human precursor miRNAs with perfect or near-perfect sequence identity may be considered to be likely functional in other primates. The computational identification of homologs with less similar sequence, instead, requires further evidence to be provided.

## Background

MicroRNAs (miRNAs) constitute a class of short endogenous non-coding RNA (ncRNA) sequences which directly function as negative regulators of gene expression at the post-transcriptional level in multicellular eukaryotes (see e.g. [1-3] for reviews). The ~70 nt long precursor of animal miRNAs (pre-miRNA) forms a typical hairpin-like stem-loop structure. The contained mature miRNA is only ~22 nt long and binds to complementary target sites in the untranslated region (UTR) of messenger RNA. Perfect base-pairing is found only for a 6-8 nt long *seed *region located at the 5' end of the miRNA. As a result, one miRNA may at least theoretically target hundreds of genes.

Comparative approaches to discover miRNA genes [4,5] rely on sequence homology to known miRNAs [6], sequence profiles [7], characteristic secondary structure features and/or evolutionary conservation among different species [8-12]. Some approaches use both sequence and secondary structure conservation to known miRNA precursors [13,14]. Berezikov *et al*. [15] use phylogenetic shadowing to derive a general conservation profile from miRNA precursor sequences of 10 primate species which is used to search for new miRNAs. *Ab initio *approaches are able to discover miRNAs in a genome without using sequence homology or conservation (see e.g. [16] and references therein).

Three non-human primate genomes have been fully sequenced and are publicly available, including rhesus monkey (*Macaca mulatta*), chimpanzee (*Pan troglodytes*), and orangutan (*Pongo pygmaeus*). While for the first two species genome-wide comparative miRNA studies have been published recently [17,18], the current list of miRNAs reported in miRBase [19] (most found in [15]) is still largely incomplete and comprises only 84 sequences. According to recent estimates supported by both genetic and fossil evidence [20], divergence of the human and ape (chimpanzee) lineages occurred about 6 million years ago (mya), orangutan and African apes diverged about 14 mya from their common ancestor, and hominoids and Old World monkeys (like rhesus macaque) about 23 mya.

## Methods

The comparative method favored in this study uses various sequence- and structure-based filters to find miRNA homologs. A combination of multiple filters not only captures more diverse aspects of miRNAs, but allows lower thresholds (lower specificity) to be used for each individual filter. This again is essential for detecting homologs that are more distant (in sequence) and allows a broader selection of more different subtypes of miRNAs.

Furthermore, different filters and thresholds are applied for accepting or rejecting a miRNA candidate which excludes a (small) third set of undecided predictions. This is to increase the confidence in both positive and negative predictions, i.e., to better control the number of false positives and false negatives.

### Homology-based analysis

The genomes of the three non-human primates were downloaded from the Ensembl database (release 50, http://www.ensembl.org). The currently known miRNAs in human were retrieved from the miRBase database [19] (release 12.0, http://microrna.sanger.ac.uk) and comprise 695 hairpin sequences and 692 different mature sequences. Many miRNAs in miRBase have been identified computationally in homology studies. The human hairpin sequences were aligned against the three primate genomes using NCBI BLAST [21] (offline version 2.2.18) with parameter settings -G 1 -E 1 -F F. Among various settings tested here, this has been found to increase the number of detected precursor homologs, compared to the standard settings.

In a second-level BLAST analysis we check the conservation of mature miRNAs by aligning all mature sequences known in human against the precursor sequences predicted in the other primates. Because of their small size, some query sequences did not produce a BLAST hit or the alignment was incomplete. In these few cases the alignment had to be manually corrected and was extended to the length of the query sequence.

### Secondary structure analysis

Structure folding, secondary structure sequence, and minimum free energy (MFE) of miRNA precursors are calculated by RNAfold from Vienna Package 1.6 [22]. The absolute *structure distance d*, the *percent structure distance P*_*d*_(*p*_1_, *p*_2_) and the *percent structure identity *(or similarity) *P*_*id*_(*p*_1_, *p*_2_) are computed between the secondary structure sequences (in dot-bracket format) of two precursors *p*_1 _and *p*_2_:(1)

All distances are based on the Levenshtein distance or string edit distance *d*_*edit *_[23] which is the minimum number of point mutations needed to transform one sequence into the other. By subtracting the length difference in Equation 1 we reduce its influence on the overall distance.

### Filtering microRNA homologs

Multiple filtering steps are to be passed by a candidate sequence to be *accepted *as a homolog of a human miRNA precursor. Only one (the best) BLAST hit is selected and processed.

(1) *Precursor sequence filter*: minimum 85 percent sequence identity over an alignment length of at least 95 percent

(2) *Structure sequence filter*: minimum 85 percent identity in secondary structure sequence

(3) *Hairpin filter*: minimum 15 base pairs in the stem arm and only one terminal loop

(4) *Seed filter*: no mutations in the seed region of the mature sequence

A cascade of Perl scripts makes the filtering process fully automatic. The selection of thresholds is partly motivated by the comparative analysis in Section *Results and discussion*. An absolute maximum of -10 kcal/mol is imposed on the MFE of a hairpin structure. This is the highest value found among known human miRNAs. The same applies to the required minimum of 15 base pairs.

The seed region is expanded to positions 2-9 [24] (from the 5' end) and extracted from each human mature miRNA. The 8mers are aligned against the mature homologs using perfect matching by Perl regular expressions.

A miRNA candidate (best BLAST match) is *rejected*, instead, and a homolog is said to be not exiting in a genome if the sequence identity drops below 70 percent.

## Results and discussion

### Comparative sequence and structure analysis

**Figure 1:**
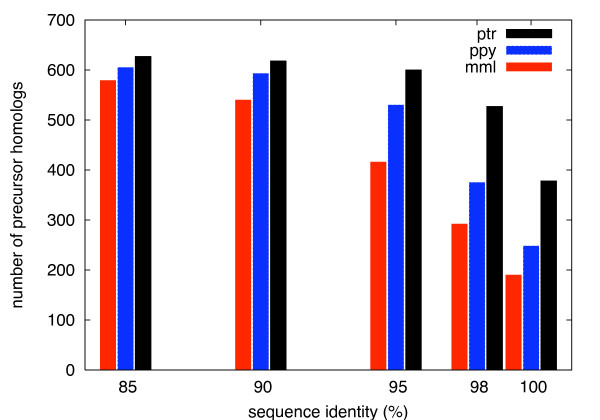
**Sequence similarity**. Number of sequence homologs of human pre-miRNAs found in rhesus macaque (mml), orangutan (ppy), and chimpanzee (ptr) using different minimum thresholds of percent sequence identity.

Figure 1 compares the number of precursor miRNAs predicted in the three non-human primates when filtering with different minimum thresholds of sequence identity. At a threshold of 85 percent, the number of pre-miRNAs begins to converge and is nearly the same in all genomes. With higher thresholds the total numbers of predictions decrease while the differences between species increase. Only 40.8 percent of the precursors are 100 percent identical between human and orangutan (ppy), compared to 60.3 percent between human and chimpanzee (ptr). It is interesting to see that above 95 percent identity the number of homologs found in orangutan is closer to the level of rhesus macaque (mml) than of chimpanzee. This could mean that, on the miRNA level, the evolutionary distance to human is actually more similar for orangutan and rhesus. It remains unclear, however, how far these figures are influenced by differences in sequencing quality between the non-human primate genomes.

miRNA homologs with perfect (100 percent) or near-perfect (around 98 percent) sequence identity allow us to assume that these are likely functional (as in human). Candidate sequences with less but more than 85 percent similarity - true for 38 percent of the precursor homologs found in orangutan - require the verification of more miRNA features.

**Figure 2:**
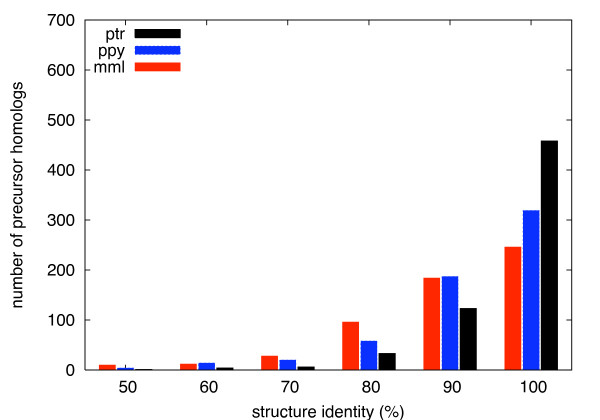
**Structure similarity**. Frequency distributions of pre-miRNA homologs over percent structure identity to human. Percent bins include all structures with ≥ *x *and *< x *+ 10 percent identity.

One important aspect is how well the structure of a miRNA precursor is preserved. Already a few nucleotide mutations can imply large structural changes or even disrupt the hairpin structure completely (see below). In Figure 2 frequency distributions of precursor homologs are plotted over the percent structure identity. As defined in Section *Methods*, this is calculated between the predicted secondary structure sequences. The vast majority of structures meets a minimum requirement of 80 percent identity. For primate species more closely related to human, this distribution is more shifted towards 100 percent. 73.0 percent of the precursor homologs found in chimpanzee show no change in structure compared to human. For the most part this is due to a 100 percent identical sequence (see Figure 1). This number compares to only 52.7 percent in orangutan and to 42.5 percent in rhesus monkey. Again, the distributions between orangutan and rhesus macaque are more similar than between orangutan and chimpanzee.

**Figure 3:**
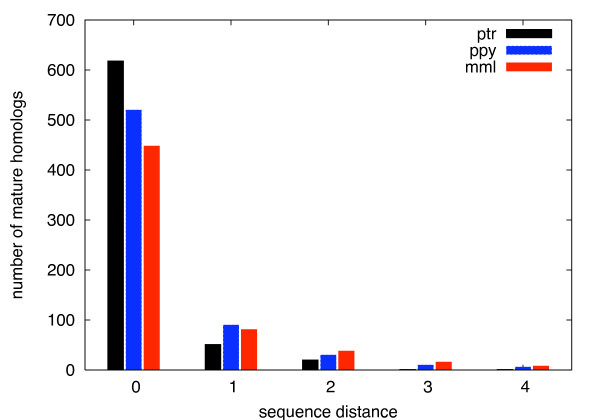
**Mature sequence conservation**. Frequency distributions of mature miRNA homologs over sequence distance to human (in number of nucleotide mutations).

Figures 3 contains the frequency distributions of nucleotide mismatches (including deletions and insertions) in the mature subsequence. First, it demonstrates that the total number of single nucleotide mutations in mature miRNA homologs varies between 0-2 only - with few exceptions - and, second, that the prevailing number of mature sequences - 89.4/79.1/75.7 percent for ptr/ppy/mml - is identical to the human counterpart. Mutations in the mature region are, obviously, most important for explaining phenotypic differences between humans and primates. Except for requiring a 100 percent conservation of the seed region, we do not further limit the number of mutations in the mature sequence. Because miRNA-target binding shows near-perfect complementarity, the seed is less variable such that mutations are less likely under positive selection.

**Figure 4:**
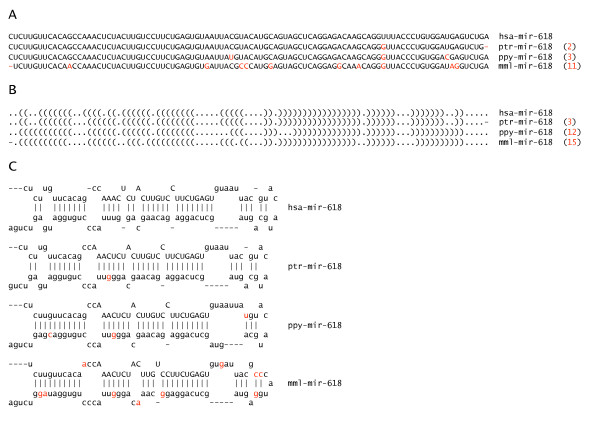
**Example**. (A) Multiple sequence alignment of pre-miRNA *mir-618 *for human (hsa) and three non-human primates. Different nucleotide positions are highlighted in red. Distances to human are given in parenthesis. (B) Corresponding secondary structure sequences in dot-bracket notation. Base pairs are represented by complementary parentheses and non-pairing bases by dots. (C) Corresponding stem-loop structures. Mature sequences are in capital letters.

As an example, the multiple sequence alignment for pre-miRNA *mir-618 *is given in Figure 4 together with the corresponding secondary structure sequences and stem-loop structures. Absolute distances to the human version (hsa) are given in number of point mutations. In case of orangutan, only a few nucleotide mutations imply a significantly higher number of local changes in the structure sequence. The global structure is, however, much less affected. Actually, the stem region - including the mature sequence - is the same as predicted for chimpanzee. In case of rhesus macaque, on the other hand, the basic stem-loop structure is still preserved even with many nucleotide mutations.

### MicroRNA gene identification in orangutan

Lists of positive and negative predictions from our analyses are provided in the supplementary material (see Section *Additional files*). Additional file 1 contains all 605 homologs of human precursor miRNAs found for orangutan, including 77 sequences which are already known (i.e. in miRBase). 18 homologs are identical or have an overlapping genome location with another miRNA. This leaves 510 newly discovered miRNAs in total.

Besides known miRNAs, candidates are marked in Additional file 1 that pass various other filters (see Section *Methods*). This allows a flexible combination of filtering criteria, including those derived from the precursor structure or the mature sequence. 526 orthologs (from 605) remain after applying the hairpin filter and 494 after the seed filter. Here, we also utilize the existence (detection) of a miRNA homolog in more than one primate species (besides human). This is to improve the reliability of predictions and helps to reduce the effect of possible sequencing errors. In our setup, 499 human precursor miRNAs are found to have a homologous sequence in all three primates. 563 miRNAs are conserved in both chimpanzee and orangutan, and 530 are shared between orangutan and rhesus macaque.

Additional file 2 lists all homologs of human mature miRNAs found in the orangutan precursors. The 682 entries include homologs of both 5' and 3' miRNAs, some originating from the same precursor. 611 human mature miRNAs are conserved in at least one orangutan precursor, resulting in 624 different sequences.

### Identification of lineage-specific microRNAs

Another question of interest is which and how many miRNAs are lineage- or species-specific. Our analysis especially supports the identification of human-specific miRNAs. Since we cannot completely exclude the possibility that some homologs may not be found because of erroneous or incomplete genome assembly, we require the negative prediction of a miRNA to be confirmed by our method in at least two of the three non-human primate genomes at hand. Additional file 3 lists all 35 homologs which are missing in this way. 12 human miRNAs could not be identified in any of the three primate genomes and, thus, are the most likely to be human-specific. These in particular may be responsible for phenotypic differences between human and non-human primates, i.e., may help to explain what makes us human.

Sequence and structural similarities to a human miRNA are strong indications for a putative homolog to be transcribed and functional. Nevertheless, the expression levels of both miRNAs may differ due to alterations in the specific regulatory pathway that controls their expression. In addition, the regulatory effects, i.e., the selection and expression of target genes, may be significantly different. This is due to a fast evolution of miRNA binding sites [25] which led to many lineage- or species-specific sites and is just as responsible for what makes us different from other primates.

## Conclusion

In this comparative study we searched the genomes of three non-human primates for miRNAs. The applied prediction algorithm (outlined in Section *Methods*) verifies multiple criteria based on similarities to known human miRNAs in sequence and structure to detect both closely-related and more distantly-related homologs. The parallel analysis allows, in particular, the prediction of a miRNA in multiple species to be used as an additional filter. In return, it provides some support for the configuration of the method, i.e., for the parameter settings (thresholds) and filter definitions used here. The other results of this study may be summarized as follows:

(1) A thorough and comprehensive search for novel orangutan miRNAs. More than 500 putative miRNA genes have been identified, where the precursor sequence is at least 85 percent identical to its ortholog in human.

(2) Both sequence distances and structure distances to human miRNAs have been found to be more similar for orangutan and rhesus macaque than for orangutan and chimpanzee, indicating a more similar evolutionary distance to human on the miRNA level.

(3) The proportion of identical or nearly identical precursor sequences with human has been found relatively small for all three primate species, considering the evolutionary distances and compared to the mature sequences. Only about 40 percent are the same in human and orangutan.

(4) Identification of common and lineage-specific miRNAs. 499 miRNA sequences are conserved in all primates investigated here. 35 human miRNAs have not been found in at least two non-human primate genomes and some of which might actually be human-specific.

## Competing interests

The author declares that he has no competing interests.

## Authors' contributions

MB is the only author of this manuscript. He designed and carried out the study, wrote the manuscript, and approved the final version.

## Supplementary Material

Additional file 1 **Orangutan miRNA genes**. Table of human pre-miRNA homologs found in orangutan with chromosome number, position, strand orientation, and precursor sequence. Candidates are marked which (1) pass the hairpin filter or (2) the seed filter, (3) are *fully *conserved in human, (4) are predicted in chimpanzee or (5) rhesus macaque, or (6) are already known, i.e., contained in miRBase. Identifiers of miRNAs with the same (overlapping) location as another miRNA are in parenthesis. Click here for file

Additional file 2 **Mature orangutan miRNAs**. Table of human miRNA homologs found in the precursor sequences predicted for orangutan (Additional file 1), including relative position and mature sequence. Click here for file

Additional file 3 **Human-specific miRNA candidates**. Table of human miRNAs which are missing (not found) in at least two non-human primate species. Non-marked miRNAs mean negative predictions. Click here for file
